# Time‐Dependent EDTA Effect on Leukocyte Differential Count and Morphology Compared to Blood Smear Made by Direct Finger Prick

**DOI:** 10.1111/ijlh.14498

**Published:** 2025-05-16

**Authors:** Anna Irding, Filip Landgren, Elisabeth Aardal

**Affiliations:** ^1^ Department of Clinical Chemistry Linköping University Linköping Sweden; ^2^ Department of Biomedical and Clinical Sciences Linköping University Linköping Sweden

**Keywords:** differential count, EDTA, leukocyte morphology, leukocytes

## Abstract

**Introduction:**

The impact of ethylenediaminetetraacetic acid (EDTA) on leukocyte count and morphology has led to the current recommendation that smears made from EDTA blood should be prepared within 4 h of sampling. However, previous studies have only been performed on smears with normal cell counts, and smears made from K_2_EDTA blood have not been studied in comparison with smears made from direct finger prick. The aim of this study was to investigate the time limit for performing a morphological assessment on smears from EDTA blood without compromising the results.

**Methods:**

Blood samples from 37 patients with known or suspected abnormal leukocyte morphology were selected, with smears from direct finger prick used as controls. Smears were prepared from the original EDTA tube every 4 h up to 24 h after sampling. The smears were evaluated for differential count, morphological assessment, and smear quality scoring.

**Results:**

No significant difference in cell count was observed on smears made up to 12 h after sampling for neutrophils (*p* = 0.430), lymphocytes (*p* = 0.080), monocytes (*p* = 0.948) eosinophils (*p* = 0.398), basophils (*p* = 0.460), and blast cells (*p* = 0.239) compared to smears made by finger prick. However, smears prepared later than 8 h postsampling showed a significant increase in morphological changes.

**Conclusion:**

A manual differential leukocyte count can be reliably performed on smears from EDTA blood within 12 h, whereas a comprehensive morphological assessment requires smears to be prepared within 8 h of sampling.

## 1 Introduction

The examination of peripheral blood by means of a complete blood count (CBC) is an important tool in the diagnosis of suspected hematological disorders. If the CBC analysis shows abnormal results, it may trigger an additional differential leukocyte count based on set criteria. In many clinical situations, the automatic leukocyte differential count provides sufficient information, but there are situations where a manual morphological evaluation is critical, such as in acute promyelocytic leukemia, where a prompt diagnosis is essential for life‐saving treatment, and a manual differential count often provides the necessary information to initiate therapy [1]. More commonly, the manual differential count plays a key role in providing diagnostic guidance and monitoring treatment outcomes in hematological malignancies.

K_2_EDTA is the preferred anticoagulant for hematological testing, valued for its ability to preserve cellular components and blood cell morphology. However, the impact of EDTA on leukocyte morphology over time has been addressed in a number of publications [2, 3, 4, 5, 6, 7, 8, 9]. While leukocyte counts can remain stable for up to 24 h at room temperature, morphological changes such as nuclear swelling, chromatin disintegration, and loss of cytoplasmic granulation have been reported to begin within half an hour of sampling in EDTA tubes. Vacuolization may also occur, and over time, nuclear alterations may create a cloverleaf appearance. These in vitro changes could falsely suggest a reactive or pathological process, potentially leading to unnecessary investigations and inappropriate patient care. Therefore, to minimize the risk of possible misclassification, the International Council for Standardization in Hematology (ICSH) recommends preparing blood smears within 4 h of sample collection, or within 6 h if kept at 4°C, to avoid morphological changes [4]. Similarly, the External Quality Assessment organisations in the Nordic countries (Equalis, Noklus) recommend that smears made from EDTA blood should ideally be prepared within 4 h from sampling, but do not clearly state a recommended temperature for sample storage [10, 11].

It is challenging to find studies that detail the time‐dependent effects of EDTA on leukocyte morphology that have influenced current recommendations. To the best of our knowledge, the effects of K_2_EDTA on leukocyte differential counts and morphological assessments have not been investigated in a study population that includes samples with abnormal leukocyte counts and abnormal leukocyte morphology in comparison to blood smears prepared from direct finger prick samples.

In our laboratory, the standard procedure is to perform a manual leukocyte differential with complete morphological assessment on smears made directly from a finger prick, to avoid any possible effect of EDTA. However, this procedure necessitates resampling when an automated differential count, flagged for suspected abnormal morphology or presence of immature cells in venous blood smears, prompts a manual review. Although this approach avoids the risk of misinterpretation due to EDTA‐related changes on leukocyte morphology, it also results in patient discomfort and delays in reporting results.

The aim of this study was to evaluate the effects of K_2_EDTA on leukocyte differential counts and morphology over a 24‐h period by comparing venous blood smears with those obtained from capillary finger prick samples. Morphological changes in erythrocytes and platelets were not included in the scope of this study.

The objective was to determine whether a blood smear from venous K_2_EDTA blood prepared within a defined timeframe after sampling could replace an additional blood smear from a capillary finger prick sample without compromising the accuracy, thereby reducing the overall turnaround time.

## 2 Methods and Material

### 2.1 Study Design

The study was conducted as a blinded comparative study to evaluate the impact of ETDA on blood smears in terms of leukocyte differential count and morphological assessment at the Department of Clinical Chemistry, Linköping University Hospital. All samples were anonymised, relabelled, and the time of smear preparation was masked to the examiner.

### 2.2 Sample Preparation

In this study, 37 blood samples were consecutively selected from the clinical routine at the Department of Clinical Chemistry, Linköping University Hospital, between December 2021 and April 2022. These samples were originally ordered for a manual leukocyte differential count by finger prick method. Each included sample was accompanied by a K_2_EDTA tube (Becton, Dickinson and Company, Plymouth, UK) for total leukocyte count, containing sufficient sample volume to allow repeated blood smears over 24 h. All samples arrived at the laboratory within 1 h of collection. At our laboratory, a manual differential count by finger prick is only ordered in cases of suspected hematological pathology or for follow‐up of a known hematological disease. Alternatively, a full morphological assessment may be requested based on laboratory recommendations if morphological abnormalities are detected in the differential count from venous blood.

The supplementary K_2_EDTA tubes were stored at room temperature for repeated smear preparation every 4 h from the time of blood draw (0 h) up to 24 h. Blood smears were prepared according to the laboratory procedure; 5 μL of well mixed blood was spread on a glass slide with either Cellavision Hemaprep (Cellavision AB, Lund, Sweden) or Molek Diffsmear DS14 (Molek AB, Årsta, Sweden). The smears were stained with May‐Grünwald Giemsa (Sigma‐Aldrich, Darmstadt, Germany) with a Molek Diffstainer (Molek AB, Årsta, Sweden).

### 2.3 Methods

The group of observers consisted of nine experienced biomedical scientists, whose competence is regularly assessed using the CellaVision Proficiency Software and the Equalis external quality control program for visual cell classification. Two different observers were randomly assigned to evaluate each smear by counting 100 leukocytes each under oil immersion light microscopy at ×1000 (×10 ocular and ×100 objective). The mean count for each cell class was calculated together with a morphological assessment. There were no clearly defined criteria for interobserver agreement, but in cases where a substantial discrepancy between two observers was noted, affecting the outcome for one or more cell classes, or when the morphological assessment was difficult, additional observers were consulted, and the mean of these counts was used. In 3 of the 37 samples, more than two observers were involved in the assessment at a single time point (*n* = 2), or at two time points (*n* = 1). The corresponding smear obtained by finger prick method was used as a reference for each sample at the different time points during data compilation, but the association between the smears was masked to the examiners.

Furthermore, the quality of the smears in terms of observed EDTA‐related changes in leukocyte morphology was assessed at each time point using a scoring system; “4”—very good quality with no difficulty in assessing differential count and morphology; “3”—adequate quality for both differential count and morphology assessment; “2”—adequate quality for a differential count, but inadequate for morphology assessment; “1”—poor quality, unsuitable for either differential count or morphology assessment. Half points were allowed if the quality was considered to be between two levels. Since the quality of the finger prick smears in the original sample group had not been evaluated, an additional 20 smears made from direct finger prick were randomly selected from the clinical routine and evaluated using the same scoring system. This was done in order to ensure consistency and reliability in the evaluation process.

### 2.4 Statistical Analyses

The residual analysis showed a skewed distribution for the differential count for all cell types as well as the quality score. Therefore, a nonparametric approach was chosen for further analysis. The Friedman test was used to compare cell counts at different time intervals. For cases that had a significant difference over time, post hoc analyses were performed using Wilcoxon signed‐rank tests with Bonferroni–Holm's correction. The Kruskal–Wallis test was used to test for quality score at the different time points and Dunnett's test was used as a post hoc test for significant differences in the median score at the different time points compared to smears prepared at 0 h. Differences were considered statistically significant at *p*‐values less than 0.05.

All statistical analyses were performed in R Studio 2021.09.0 (R version 4.03). The rstatix package was used for the Friedman test. The DescTools package was used for Dunnett's test. The ggplot2 package was used for data visualization.

## 3 Results

### 3.1 Missing and Excluded Data

Of the 37 samples, two were initially excluded: one due to the absence of smears at two time points and the other due to difficulty in distinguishing between blast cells, atypical mononuclear cells, and monocytes at all time points, including the reference smear, and was therefore not considered to be caused by a storage effect. For nine samples, differential counts were missing at one or more time points due to poor smear quality, preventing full statistical analysis at those specific time points.

### 3.2 Agreement of Differential Count

Initial statistical analysis was performed at 0, 4, and 12 h as these were the only time points with complete differential count results for all included samples (*n* = 35). Results for other time points were incomplete, with missing data for two samples at 8 h, one sample at 16 h, five samples at 20 h, and six samples at 24 h. The results from the different time points for each cell class are presented in Table 1. Blast cells were detected in 12 of the 35 samples, with the proportion exceeding 2% in 10 of these samples (Table 1).

**TABLE 1:** Median values for most frequent cell classes observed in the study samples at various time points, with ranges (lowest to highest) provided in parentheses.

Cell class	Finger prick (*n* = 35)	0 h (*n* = 35)	4 h (*n* = 35)	12 h (*n* = 35)	24 h (*n* = 29)
Neutrophils	6.12	6.47	5.94	5.61	3.87
(× 10^9^/L)	(0.00–56.14)	(0.00–48.40)	(0.00–40.66)	(0.01–45.98)	(0.01–46.46)
Lymphocytes	2.06	1.83	1.98	1.76	2.29
(× 10^9^/L)	(0.51–15.50)	(0.40–13.43)	(0.39–12.64)	(0.47–11.95)	(0.44–12.64)
Monocytes	0.56	0.60	0.46	0.67	0.67
(× 10^9^/L)	(0.00–16.19)	(0.01–16.53)	(0.01–16.79)	(0.02–16.01)	(0.00–22.21)
Eosinophils	0.08	0.07	0.19	0.20	0.06
(× 10^9^/L)	(0.00–1.14)	(0.00–0.76)	(0.00–1.45)	(0.00–1.09)	(0.00–1.27)
Basophils	0.00	0.01	0.00	0.00	0.00
(× 10^9^/L)	(0.00–0.75)	(0.00–1.50)	(0.00–0.75)	(0.00–0.64)	(0.00–0.75)
Blast cells	0.00	0.00	0.00	0.00	0.00
(× 10^9^/L)	(0.00–198.74)	(0.00–199.82)	(0.00–208.51)	(0.00–207.43)	(0.00–204.17)

Friedman's test showed no significant difference in cell counts up to 12 h compared to the initial count on smears made by finger prick for segmented neutrophils (*p* = 0.430), lymphocytes (*p* = 0.080), monocytes (*p* = 0.948), eosinophils (*p* = 0.398), basophils (*p* = 0.460), and blast cells (*p* = 0.239). As the data from the original sample group (*n* = 35) did not show significance up to 12 h, further analysis on the available data at 24 h (*n* = 29) was performed using the Wilcoxon signed‐rank test. The cell counts were not significantly different at 24 h compared to results from the finger prick smear results for lymphocytes (*p* = 0.051), monocytes (*p* = 0.081), eosinophils (*p* = 0.902), basophils (*p* > 0.999), and blast cells (*p* = 0.444). However, a significant decrease in segmented neutrophils was observed at 24 h in comparison to the initial smear obtained by finger prick (*p* < 0.001). To ascertain whether the total neutrophil count was affected over time, a new calculation was performed adding the band neutrophil count to the segmented neutrophil count for all samples (*n* = 29) with a statistically significant (*p* < 0.001) decrease also for the pooled neutrophil group on smears performed 24 h after sampling.

### 3.3 Quality of the Smears

**FIGURE 1:**
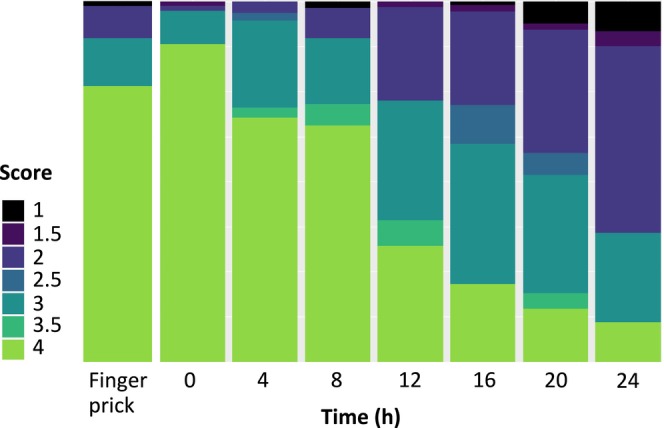
Distribution of the quality scores at each time point. 4: *Very good quality with no difficulty assessing differential count and morphology*; 3: *adequate quality for both differential count and morphology assessment*; 2: *adequate quality for a differential count, but inadequate for morphology assessment*; 1: *poor quality, unsuitable for either differential count or morphology assessment*.

Smears prepared at 12 h or later presented a significantly lower quality score (*p* < 0.001) compared to smears prepared from EDTA blood immediately after sampling. There was no significant difference in quality observed at 4 h (*p* = 0.714) and 8 h (*p* = 0.080) compared to 0 h. The additional group of 20 smears prepared from finger prick samples demonstrated a mean quality score of 3.4 [CI: 3.0–3.8], comparable to the results observed in the original study group up to 8 h (0 h: 3.8 [CI: 3.6–4.0]; 4 h: 3.5 [CI: 3.3–3.8]; 8 h: 3.3 [CI: 3.0–3.6]). At 12 h, the score decreased to 2.8 [CI: 2.6–3.1] and further declined to 2.1 [CI: 1.8–2.3] at 24 h (Figure 1).

### 3.4 Morphological Changes

**FIGURE 2:**
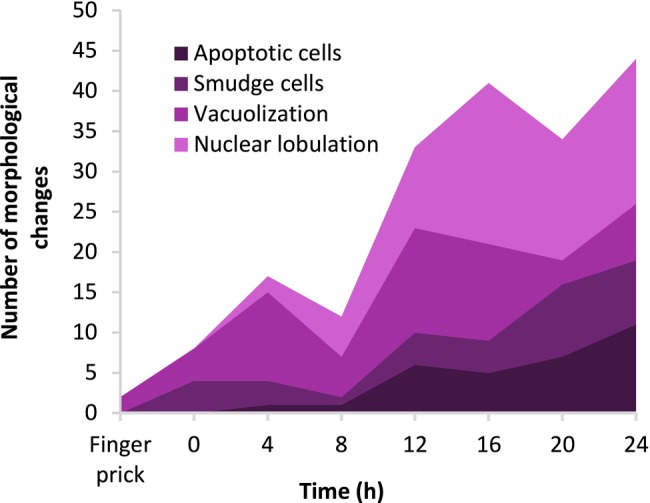
Frequency of reported morphological changes for leukocytes related to EDTA at the different time points in the study sample.

**FIGURE 3:**
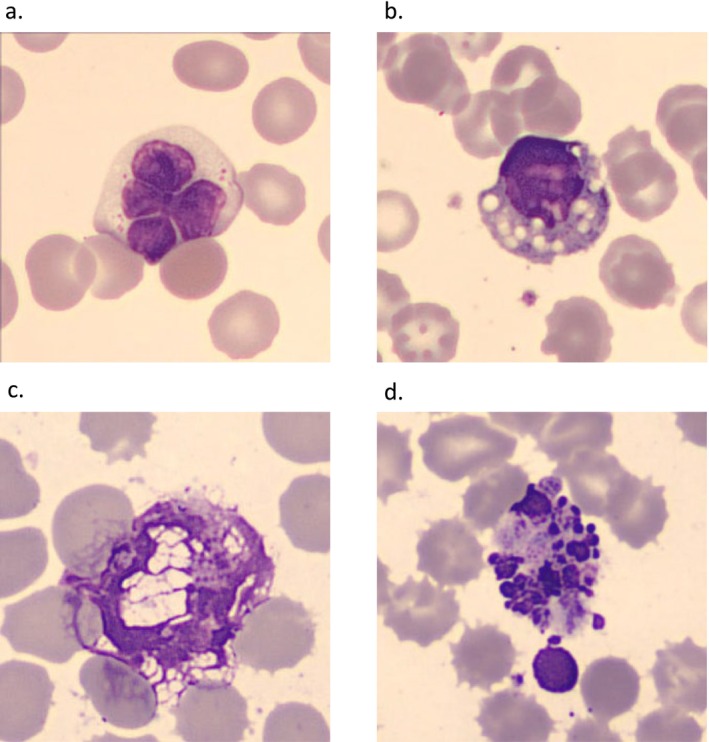
EDTA‐related morphological changes for leukocytes after storage in room temperature; (a) lymphocyte with nuclear lobulation, (b) mononuclear cell with cytoplasmic vacuoles, (c) smudge cell, and (d) neutrophil granulocyte in apoptosis.

A morphological evaluation of known EDTA‐related changes was conducted to validate the quality score results. The most notable changes observed in the study included nuclear lobulation and cytoplasmic vacuolization. In addition, an increasing number of apoptotic cells with pyknotic nuclei were observed over time (Figures 2 and 3).

Although not within the scope of the study, a gradual increase in the presence of echinocytes and large platelets was also noted over time.

## 4 Discussion

Microscopic evaluation of peripheral blood smears is essential in the diagnosis of hematological disorders, with particular emphasis on the assessment of dysplastic changes. The impact of EDTA on leukocyte morphology and its potential to cause misinterpretation has been addressed in several publications [2, 3, 5, 6, 7, 8, 9], resulting in the current guidelines from ICSH [4] and Equalis [10] recommending that EDTA blood smears should be prepared within 4 h from sampling.

Vives‐Corrons et al. [6] demonstrated that significant morphological changes, such as vacuolization, Pelger–Huet‐like forms, and neutrophil degranulation, occur within 4 h of sampling at both room temperature and 4°C. The authors concluded that their findings support the ICSH recommendation; however, the study only evaluated samples at 0, 4, 12, and 24 h, and morphological changes were recorded based on whether more or less than 50% of the examiners observed them.

Other studies have reported varying time frames for acceptable smear preparation. Houwen [7] stated that EDTA‐anticoagulated venous blood is acceptable for smear preparation, with best results obtained when smears are prepared within 2 h, but short‐term storage up to 8 h at room temperature is acceptable. Similarly, Kennedy et al. [8] found no significant changes in leukocyte morphology within 5 h after sampling, the maximum time frame studied. While they noted morphological changes such as indentation and vacuolization, these were not considered to interfere with cell identification or distinguishing abnormal cells. However, the authors mentioned the need for further testing on pathological samples.

Despite these findings, the effect of K_2_EDTA on reactive or pathological cells remains largely unexplored, as most studies have focused on samples with normal leukocyte counts and morphology. To address this gap, the present study aimed to elucidate the effect of K_2_EDTA over 24 h on leukocyte differential counts and morphology, including both normal and pathological leukocytes, comparing venous blood smears to those obtained directly from capillary finger pricks.

Our results confirm previous findings [3, 5, 6, 8] that EDTA‐related morphological changes increase over time, making reliable morphological assessment more difficult as smear quality declines. To eliminate potential confounding variables related to smear preparation, we standardized smear preparation using semiautomated devices. Our findings of an increasing number of smudge cells and apoptosis‐related changes suggest that cells become increasingly susceptible to the mechanical stress during smear preparation over time, particularly after 12 h. Neutrophil granulocytes were found to be more vulnerable than other cell classes, showing a significant reduction in smears prepared after 12 h. However, it remains unclear whether this fragility is solely due to the effects of EDTA or whether it is related to metabolic damage over time.

In contrast to previous studies [6, 8], this study included samples with both normal and pathological leukocyte distribution and morphology, confirmed by reference smears from direct finger prick. Our findings show that the previously described morphological changes and increased fragility over time are also present in pathological leukocytes. An important clinical concern remains whether these EDTA‐induced morphological changes could lead to misclassification of cells as reactive or dysplastic, potentially causing mistreatment. For instance, myelodysplastic syndrome might be suspected due to findings such as hypogranulation, pseudo Pelger–Huet cells, or nuclear irregularities, all of which could also be attributable to EDTA‐induced changes.

This study further examined how long the technical quality of a blood smear made from K_2_EDTA blood could be maintained after sampling, using a subjective quality assessment for each smear. The results indicate that reliable morphological assessment is possible when smears are prepared within 8 h after sampling.

As manual microscopic examination is highly dependent on the individual examiner, it is important to consider between‐examiner variation when interpreting study results, even with highly experienced laboratory technicians. The current Equalis guideline [10] recommends two examiners count and assess 100 cells per preparation, with the mean values for the different cell classes being calculated. In clinical practice, the examiners may discuss difficult cases, sometimes involving a third examiner. In this study, each examiner conducted an independent review of the smears, and the mean of the two independent assessments was calculated a posteriori. This approach may have resulted in discrepancies in the results, particularly in cases with pathological cells, where the examiners had no opportunity to discuss their findings and, moreover, no clinical information was provided. In addition, the examiners were aware that the smears were prepared from EDTA blood, whereas, according to local laboratory protocols, EDTA smears are not normally used for morphological assessment. This awareness may have introduced some bias and increased between‐examiner variation compared to what would be expected in clinical practice. However, due to the study design in which two examiners were randomly selected from a group of nine for each sample, it was not possible to account for this variation.

The investigation of erythrocyte and platelet morphology was not within the scope of this study, which primarily focused on leukocyte morphology. However, in cases of poor technical smear quality after 12–24 h, common findings involved not only morphological changes in leukocytes but also an increased occurrence of echinocytes and large platelets. These observations suggest that, similar to leukocytes, erythrocytes and platelets undergo time‐dependent changes that may have clinical relevance, a matter that requires further investigation.

A limitation of our study is its single‐center design, which may affect the generalizability of the results. The samples came from a specific patient group under investigation or treatment for hematological disorders, including both normal and pathological leukocyte distributions, but did not represent all patient groups and diagnoses where a morphological assessment is of importance.

## 5 Conclusions

Consistent with current recommendations [4, 10, 11], smears prepared within 4 h of venous sampling in K_2_EDTA provided sufficient quality for differential leukocyte counting. Furthermore, our results suggest that the time limit between sampling and smear preparation could potentially be extended to 8 h for morphological assessment and up to 12 h for differential counts, even in the presence of dysplastic cells.

This study adds valuable knowledge regarding the time‐dependent effects of EDTA on leukocytes and suggests that reliable results for both differential counts and morphological assessment may be achievable for a longer period than currently recommended. However, confirmation through further studies with a broader range of patient populations would help validate these findings.

## Author Contributions

The study was conceptualized by A.I. and E.A. A.I. prepared the initial draft. F.L. conducted the data analysis. E.A. contributed to data interpretation and provided critical revisions. All authors have read and approved the final version.

## Ethics Statement

The study is covered by the Swedish Biobank Act and ethical approval was therefore not required. The samples were treated anonymously and used for the same purpose as specified in the original request.

## Conflicts of Interest

The authors declare no conflicts of interest.

## Data Availability

The data that support the findings of this study are available from the corresponding author upon reasonable request.
